# Main reasons and predictive factors of cancer-related emergency department visits in a Hungarian tertiary care center

**DOI:** 10.1186/s12873-022-00670-0

**Published:** 2022-06-23

**Authors:** Márton Koch, Csaba Varga, Viktor Soós, Lilla Prenek, Lili Porcsa, Alíz Szakáll, Gergely Bilics, Balázs Hunka, Szabolcs Bellyei, János Girán, István Kiss, Éva Pozsgai

**Affiliations:** 1 Department of Emergency Medicine, Somogy County Kaposi Mór General Hospital, Tallián Gyula Street 20-32, Kaposvár, 7400 Hungary; 2 Department of Emergency Medicine, Semmelweis University, Üllői Street 78/A, Budapest, 1082 Hungary; 3 Department of Oncotherapy, University of Pécs Clinical Center, Édesanyák Street 17, Pécs, 7624 Hungary; 4 Department of Public Health Medicine, University of Pécs Medical School, Szigeti Street 12, Pécs, 7624 Hungary; 5 Department of Primary Health Care, University of Pécs Medical School, Rákóczi Street 2, Pécs, 7623 Hungary

**Keywords:** (3–10) Emergency Department, Cancer, Cancer patients, Predictive factor, Emergency department visit, Hospitalization

## Abstract

**Background:**

Identifying the reasons for the Emergency Department (ED) visit of patients with cancer would be essential for possibly decreasing the burden of ED use. The aim of our study was to analyze the distribution of the demographic and clinical parameters of patients with cancer based on the reasons for the ED visits and to identify possible predictive factors for their visits.

**Methods:**

This retrospective study, carried out at a large, public tertiary hospital in Hungary, involved all patients 18 years or over, who had received a cancer diagnosis latest within five years of their visit to the ED in 2018. Demographic and clinical characteristics were collected partly via automated data collection and partly through the manual chart review by a team of experts, including six emergency physicians and an oncologist. Five main reasons for the ED visit were hypothesized, pilot-tested, then identified, including those with cancer-related ED visits (whose visit was unambiguously related to their cancer illness) and those with non-cancer-related ED visits (whose visit to the ED was in no way associated with their cancer illness.) A descriptive approach was used for data analysis and binary logistic regression was used to determine predictive factors for patients with cancer visiting the ED.

**Results:**

23.2% of the altogether 2383 ED visits were directly cancer-related, and these patients had a significantly worse overall survival than patients with non-cancer related ED visits. Age 65 or below (Odds Ratio: 1.51), presence of two more comorbidities (OR: 7.14), dyspnea as chief complaint (OR: 1.52), respiratory cancer (OR: 3.37), any prior chemotherapy (OR: 1.8), any prior immune/biological treatment (OR: 2.21), any prior Best Supportive Care/palliative care (OR: 19.06), or any prior hospice care (OR: 9.43), and hospitalization (OR:2.88) were independent risk factors for the ED visit to be cancer-related.

**Conclusions:**

Our study is the first to identify independent predictive factors of ED use by patients with cancer based on the chief cause of their visit in the Central and Eastern European region. These results may provide important information for the development of algorithms intended to identify the needs of care of patients with cancer at the ED.

**Supplementary Information:**

The online version contains supplementary material available at 10.1186/s12873-022-00670-0.

## Background

Cancer is the second leading cause of mortality in developed countries, causing a tremendous burden for both society in general and the healthcare systems as well [1]. Patients receive care from multiple providers and specialists, with a significant proportion of patients with cancer visiting the Emergency Department (ED) in the course of their illness [2, 3]. Although ED care is appropriate for the treatment of unexpected acute onset medical conditions, it is not recommended in certain instances.

For oncology patients, ED visits may indicate gaps in healthcare services, which could possibly be filled by more proactive routine cancer care or other forms of non-emergency care [4]. In addition, ED physicians are often not trained to provide adequate symptom management for patients with advanced cancer, yet, according to a recent study, the 5 most common ED diagnoses were cancer symptom-related when visiting the ED [5–8]. Since EDs are often overcrowded and patients may have longer waiting times depending on their condition’s urgency, it is questionable whether this environment is the best place of care for every patient with cancer presenting to the ED [9, 10]. Yet, patients receiving or having received oncological care tend to utilize emergency care more often, between 1 and 83%, compared to patients without cancer [2] and according to a study conducted in the US, the outcome of patients with cancer undergoing oncological treatment presenting to the ED, was also worse [10]. According to a large nationwide population-based study in South Korea patients with cancer accounted for 6.8% of all ED visits, with a high overall in-hospital mortality of 16.1% [3].

The diversity and complex care needs of patients with cancer as well as their comparatively frequent visits to the ED contrasted with the overburdened and fast-paced clinical environment of EDs has led to a number of investigations aimed at identifying the main causes and predictive factors for ED visits by patients with cancer [2]. African American race, older age, male sex, and cancer stage have been shown to possibly influence ED use [11–13], while fever, urinary complaints, malnutrition and neutropenia where some of the main reasons for the ED visit as indicated by investigations focusing on certain cancer types [14–18]. Identifying the reasons and factors leading to ED use are essential for determining and decreasing the burden of ED use among patients with cancer.

To date no Central- and Eastern European studies have been published about the incidence and characteristics of patients with cancer presenting to the ED, since most analyses have been reported from Western countries. Furthermore, no analyses have investigated the predictive factors of cancer-related ED visits in relation to the main reason for the ED visit.

Therefore, the objective of our study was to investigate the relationship between the clinical and demographic characteristics of patients with cancer presenting to a large Hungarian tertiary-care ED and the main reasons for their ED visits. It was also our aim to identify possible predictive factors of ED visits made by patients with cancer, when presenting to the ED due to the progression or complication of their cancer disease.

## Methods

### Setting

The study was carried out at a large, public tertiary hospital, the Somogy County Kaposi Mór Teaching Hospital, in Kaposvár, Hungary, which includes a level 3 Emergency and Trauma Center (ED) and a dedicated cancer center. All patients (including patients with and without cancer) with acute symptoms are encouraged to visit the ED first (as part of the single-gate system), where the patients are triaged according to the MSTR (Magyar Sürgősségi Triázs Rendszer, the Hungarian Emergency Triage System [19]), that is based on the Canadian Triage and Acuity Scale, CTAS [20], admitted and subsequently examined and evaluated. Then the patient may, on the basis of their medical condition, be either (1.) discharged home after examination or (2.) admitted to an inpatient ward of the hospital (hospitalized) or admitted to the short-term ward of the emergency department (for up to 24 hours) after which he/she is either admitted to the hospital or discharged. There is no outpatient phone triage system in Hungary, therefore all patients presenting to the ED will be admitted without prior phone triage, however patients with minor problems also have access to the 24-hour GP on-call system. The annual patient turnover of the ED is approximately 35,000 patients and 80% of the patients are over 18 years of age. The hospital, where our study was conducted, includes a dedicated cancer center with an inpatient unit, a day oncology unit and a radiotherapy unit, and is responsible for the treatment of patients with cancer in Somogy county but also accepts patients from neighboring counties.

Our study received ethical approval from the Regional Ethical Committee prior to the research procedure (Reference number: 8280-PTE2020).

### Study design

This was a retrospective study of patients with a cancer diagnosis, so with an ICD-10 code, who visited the ED in 2018. The ICD-10 is the International Classification of Diseases, 10th Revision which is the official system for assigning codes to diagnoses and procedures in the ED and in the other units of the hospital in Hungary. The ICD-10 codes used to screen and identify the patients with cancer were C0000-C9670. We included all patients with a cancer diagnosis above 18 years; and who visited the ED between 1 January - 31 December 2018; and who had received their diagnosis of cancer within 5 years of their first ED visit in 2018 or received their diagnosis of cancer latest within the study year.

The hospital’s electronic database was screened for all patients who met the inclusion criteria. In the study year of 2018, there were altogether 27,010 visits made by patients 18 years and older at the ED, from which 2383 cases were made by patients who had received an ICD-10 cancer diagnosis latest in the year 2018, thus constituting 8.8% of all adult ED visits.

A thorough chart review was carried out. First, automated data collection was performed, which included the collection of demographic data (patient’s, age at first ED visit, place of residence), date and time of the ED visit, number of ED visits per patient, visit day and visit hour category, type of cancer, type and number of comorbidities, time and date of prior oncological care, triage categorization, chief complaints, diagnosis given following ED admission, disposition (admitted to inpatient care, discharged), place of inpatient care following ED presentation and -where applicable- time of death of the patient. Types of cancer diagnoses, diagnoses of comorbidities, chief complaints and diagnoses given following ED admission were classified according to ICD-10. Since most of the time more than one ICD-10 codes were assigned to a patient per ED visit, the code listed as first (in diagnosis position one) for the ED visit was considered the primary diagnosis: the “diagnosis given following ED admission”. (In our hospital’s eMedSol database, this is unambiguously recorded as the “main diagnosis” by the emergency physician evaluating the patient.) In line with this system, similarly, if more than one cancer diagnoses were present, the code listed as first was considered the primary cancer type, if more than one symptom was listed, the code listed as first was considered the chief complaint.

Since automated data collection does not provide sufficient data when determining the underlying causes of a given ED visit (if a patient has back pain and is given an ICD-10 code for “Pain” for example, the code in itself is unable to indicate whether the back pain is caused by lumbago or the progression of bone metastases), therefore – following automated data collection – a manual chart review was carried out. A team of experts, including six specialists in emergency medicine, reviewed the medical records and determined the main reason for the ED visit. First, a hypothesis for the main reasons of the ED visits made by patients with cancer was set up by the team of emergency physicians and oncologist. Then the pilot testing of 200 patients’ data was carried out. Finally, based on the consensus of the researchers (regarding the criteria for a given ED visit) the following five main reasons for patients’ ED visits were identified (Table 1).

**Table 1:** The definitions of the 5 main reasons for the ED visits of patients with cancer

Reason for ED visit	Definition
Cancer-related ED visit	patients whose visit was unambiguously related to their cancer, i.e. who visited the ER due to the complications or progression of their cancer
Oncological care-related ED visit	patients with cancer whose visit was due to the complications/adverse events of some form of oncological care they had received prior and nearest to the ER visit
New cancer diagnosis-related ED visit	cases where a strong suspicion of cancer arose at the given ER visit, which diagnosis was subsequently confirmed
Non-cancer related ED visit	patients whose visit to the ER was in no way associated with their cancer illness
Undetermined ED visit	patients whose medical condition could be a result of either the complication/progression of their cancer or due to completely other causes (e.g. pneumonia in a patient with lung cancer and advanced COPD could be due to either of the latter two illnesses)

Subsequently, detailed review of the relevant medical records of the rest of the 2383 ED patient visits was carried out and each was individually categorized into one of the indicated five main reasons for the ED visits. Two emergency physicians reviewed the medical charts of the same patient. The process of the manual chart review included screening the database for tumorboard medical records within the study period, which thus contained most of the relevant data of the patient’s cancer from the viewpoint of our study. Medical records nearest in time to the given ED visit and from the department presumed to be related to the cancer were initially assessed for data collection. Due to the heterogeneity of the diseases, medical conditions and the patients, a general guideline for evaluation was established in which the expert reviewers took into account, among other parameters, the oncological disease, its stage, comorbidities, chief complaint and presenting diagnosis, the duration of the most recent oncological treatment and its potential spectrum of side effects. The data was then evaluated based on the professional knowledge and experience of the emergency physicians, to decide on the reason of the ED presentation. A third reviewer was consulted if there was a disagreement regarding the reason of the ED visit.

Table 2 includes the definitions and the criteria that were used for categorisation of the data. Table 3 shows the characteristics of the ED visits made by patients with cancer in 2018.

**Table 2:** Definitions and criteria for the categorisation of data used in the study

Demographic and Clinical Data	Definition/Categorisation
Age (years)	≤65 or > 65 years
Time of ED visit	
Regular clinic hours	Non-holiday weekdays Monday through Friday between 8:00–16:00,
Off-clinic hours	Weekends or holidays or weekdays 16:01–07:59
Types of Cancer (12 categories)	Colorectal cancer, Breast cancer, Gastroesophageal cancers (including cancers of the stomach and the esophagus),
	Genitourinary cancers (including all cancers of the genitourinary tract, except prostate cancer), Prostate cancer,
	Head and neck cancers, Cancers of the pancreas, liver, biliary tract and the small intestine, Respiratory (mostly lung) cancers,
	Hematological malignancies, Melanoma, Non-melanoma skin cancers, Other (including all other primary cancers excluded from the other categories and metastases).
Number of comorbidities	0, 1, ≥2
Oncological care prior to ER visit	Any type of inpatient or outpatient oncological care (BSC, palliative care, hospice) or treatment (chemo-, radio-, immunotherapy or surgery), which the patient received closest to the current ED visit’s date.
Types of Oncological care	Surgical-, radio-, chemo-, immune- or biological- and hormone treatments as well as supportive care (BSC/palliative care) and hospice
Time elapsed between the given ED visit and prior oncological care	The number of days between the first day of any form of the previous oncological care and the date of the nearest subsequent ER visit, collapsed into two categories: “≤30 days” and “> 30 days”
Triage (MSTR, Hungarian Emergency Triage System)	5, Non-urgent
	4, Less urgent
	3, Urgent
	2, Emergent
	1, Resuscitation For the purpose of the analysis, Triage level 1–4 patients were classified as „urgent” and Triage level „5″ patients as non-urgent.
Chief complaints	Main symptom or complaint of the patient, the reason the patient visited our ED. Classified according to the ICD-10 coding, were collapsed into 21 main categories based on the affected organs and/or the frequency of the given symptom as determined by the expert group.
Diagnosis given following ED admission	Diagnosis given following ED admission indicates the patient’s present disease/medical condition for which he/she visited the ED. It is the final diagnosis given by the emergency physician who evaluated the patient after ED admission.
	Classified according to the ICD-10 coding, diagnoses were collapsed into 24 main categories based on the affected organs and/or the frequency of the given symptom as determined by the expert group.
Destination from ED	Grouped into 3 categories: discharged to place of primary residence, admitted to the inpatient area or discharged against medical advice.
BSC/palliative care	Best supportive care or specialized palliative care, where available

**Table 3:**
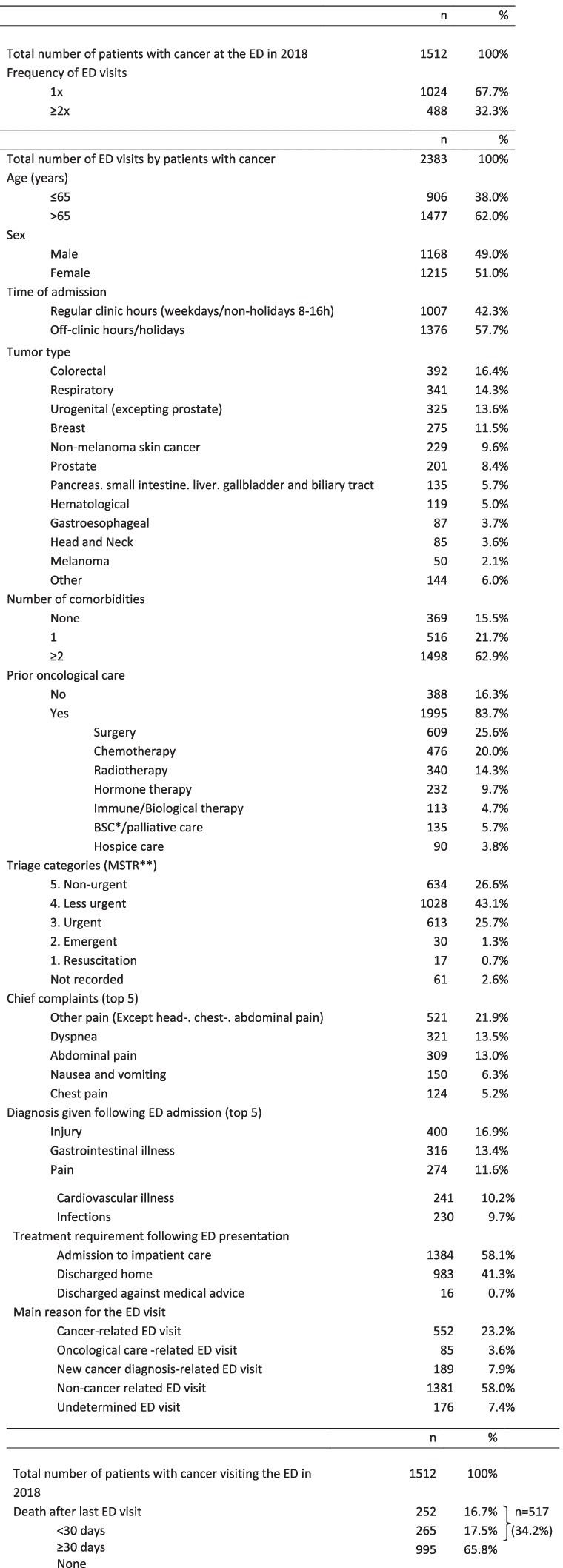
The number of ED visits by patients with cancer in 2018 (A), the demographic and clinical characteristics of ED cases (B) and the number and time of death (C)

*Best Supportive Care

**Magyar Sürgősségi Triage Rendszer (Hungarian Emergency Triage System)

The primary outcome measure for this study was the distribution of the demographic and clinical characteristics of cases across the five “main reasons for ED visits” categories. The secondary outcome measures for this study were the predictors of cancer-related ED visits of patients with cancer.

Patients were followed up for 36 months following their last ED visit and - where relevant - the death of the patients was recorded. The number of deaths within 30 days, and between 30 days and 3 years from the last ED presentation were also documented (Table 3).

### Data analysis

The data analysis framework was developed to address the research questions set for the study. Both descriptive and exploratory approaches were used. Two data sets were created: one contained the data of the 1512 patients with cancer who had attended the ED during the study period. The other data set included the total number of ED visits (2383 cases) made by the patients with cancer in the study period. The use of two datasets was necessary because a patient may have visited the ED more than once during the study period. Moreover, some of the characteristics were related to the patient (number of visits, data related to death), while others were related to the ED visit. A descriptive approach was used to assess the number of ED visits made by patients with cancer, the demographic and clinical characteristics of ED cases and the number and time of death. Contingency table analysis, Chi-squared test, and Fischer’s exact test for low case numbers were used to identify ED visits’ main reasons. Differences and associations were considered statistically significant if *p* ≤ 0.05. A survival curve, analyzed by log-rank test (*p* ≤ 0.05), was applied to examine the overall survival (OS). Binary logistic regression (backward conditional) was used to determine predictive factors for patients with cancer visiting the ED: values of independent variables indicated cancer-related or non-cancer-related reasons while the variables reported in Fig. 2 were used as dependent variables.

## Results

### 1. The distribution of demographic and clinical characteristics of patients with cancer based on the main reasons for the ED visit

We analyzed the demographic and clinical characteristics of the patients based on the reasons for their visits to the ED. Cancer-related ED visits resulted in the highest percentage of repeated ED visits, with 51.5% (*p* = 0.000) of all cancer-related visits being repeated visits (Table 4).

**Table 4:** Characteristics of patients with cancer based on the main reasons for the ED visit in 2018. A) Number of ED visits per patient based on the reason for the ED visit B) The distribution of demographic and clinical characteristics of patients with cancer based on the main reasons for the ED visit, C) Number of deaths following last ED presentation

Frequency of ED visits (*p* = 0.000)	Cancer-related ED visit *n* = 330	Oncological care -related ED visit *n* = 54	New cancer diagnosis -related ED visit *n* = 115	Non-cancer related ED visit *n* = 913	Undetermined ED visit *n* = 100	Total number of patients with cancer visiting the ED *N* = 1512
1x	160 (48.5%)	40 (74.1%)	102 (88.7%)	659 (72.2%)	63 (63.0%)	1024 (67.7%)
2-3x	132 (40.0%)	14 (25.9%)	11 (9.6%)	216 (23.6%)	33 (33.0%)	406 (26.8%)
≥ 4x	38 (11.5%)	0 (0.0%)	2 (1.7%)	38 (4.2%)	4 (4.0%)	82 (5.5%)
	Cancer-related ED visit *n* = 552	Oncological care -related ED visit *n* = 85	New cancer diagnosis -related ED visit *n* = 189	Non-cancer related ED visit *n* = 1381	Undetermined ED visit *n* = 176	Total number of ED visits by patients with cancer *N* = 2383
Sex (*p* = 0.038)						
Male	267 (48.4%)	41 (48.2%)	110 (58.2%)	654 (47.4%)	96 (54.5%)	1168 (49.0%)
Female	285 (51.6%)	44 (51.8%)	79 (41.8%)	727 (52.6%)	80 (45.5%)	1215 (51.0%)
Age (years) (*p* = 0.000)						
≤ 65	253 (45.8%)	45 (52.9%)	78 (41.3%)	475 (34.4%)	55 (31.3%)	906 (38.0%)
> 65	299 (54.2%)	40 (47.1%)	111 (58.7%)	906 (65.6%)	121 (68.8%)	1477 (62.0%)
Admission to ED during business hours (*p* = 0.311)						
Yes	219 (39.7%)	31 (36.5%)	88 (46.6%)	597 (43.2%)	72 (40.9%)	1007 (42.3%)
No	333 (60.3%)	54 (63.5%)	101 (53.4%)	784 (56.8%)	104 (59.1%)	1376 (57.7%)
Number of comorbidities (*p* = 0.000)						
0	100 (18.1%)	17 (20.0%)	46 (24.3%)	181 (13.1%)	25 (14.2%)	369 (15.5%)
1	130 (23.6%)	23 (27.1%)	47 (24.9%)	279 (20.2%)	37 (21.0%)	516 (21.7%)
≥ 2	322 (58.3%)	45 (52.9%)	96 (50.8%)	921 (66.7%)	114 (64.8%)	1498 (62.9%)
Days since previous oncological care (*p* = 0.000)						
< 30 days	165 (35.6%)	65 (78.3%)	1 (4.2%)	146 (12.4%)	49 (36.8%)	426 (22.6%)
≥ 30 days	299 (64.4%)	18 (21.7%)	23 (95.8%)	1031 (87.6%)	84 (63.2%)	1455 (77.4%)
Triage category (MSTR*) (*p* = 0.000)						
5	98 (18.2%)	16 (19.8%)	65 (34.6%)	414 (30.8%)	41 (24.1%)	634 (27.3%)
4	224 (41.7%)	46 (56.8%)	79 (42.0%)	597 (44.4%)	82 (48.2%)	1028 (44.3%)
3	198 (36.9%)	18 (22.2%)	41 (21.8%)	319 (23.7%)	37 (21.8%)	613 (26.4%)
2	11 (2.0%)	1 (1.2%)	3 (1.6%)	11 (0.8%)	4 (2.4%)	30 (1.3%)
1	6 (1.1%)	0 (0.0%)	0 (0.0%)	5 (0.4%)	6 (3.5%)	17 (0.7%)
Destination from ED (*p* = 0.000)						
Discharge	228 (41.3%)	39 (45.9%)	74 (39.2%)	957 (69.3%)	86 (48.9%)	1384 (58.1%)
Hospitalization	323 (58.5%)	45 (52.9%)	115 (60.8%)	416 (30.1%)	84 (47.7%)	983 (41.3%)
DAMA**	1 (0.2%)	1 (1.2%)	0 (0.0%)	8 (0.6%)	6 (3.4%)	16 (0.7%)
Death after last ED visit (*p* = 0.000)	Cancer-related ED visit *n* = 330	Oncological care-related ED visit *n* = 54	New cancer diagnosis-related ED visit *n* = 115	Non-cancer related ED visit *n* = 913	Undetermined ED visit *n* = 100	Total number of patients with cancer visiting the ED *N* = 1512
< 30 days	154 (46.7%)	9 (16.7%)	20 (17.4%)	47 (5.1%)	22 (22.0%)	252 (16.7%)
≥ 30 days	68 (20.6%)	8 (14.8%)	34 (29.6%)	132 (14.5%)	23 (23.0%)	265 (17.5%)
None	108 (32.7%)	37 (68.5%)	61 (53.0%)	734 (80.4%)	55 (55.0%)	995 (65.8%)

*MSTR Hungarian Emergency Triage System

**DAMA Discharged against medical advice

ED visits were more frequent among females, with ED visits by males being significantly more frequent only among undetermined cases or among cases with a new cancer diagnosis –related ED visit (*p* = 0.038). ED visits were significantly more frequent (*p* = 0.000) among patients above 65 years of age in all groups, except among oncological care-related cases. The reason for the ED visit was not significantly affected by the time of the visit, but presentation to the ED during off-clinic hours was more frequent in all groups. Patients with non-cancer related ED visits had the significantly highest proportion (66.7%; *p* = 0.000) of 2 or more comorbidities, while patients with a new cancer-related ED visit had the lowest (50.8%). Cases of oncological care-related ED visits had the highest proportion of patients with oncological care 30 days prior to the ED visit and it was the only group of patients where the majority (78.3%) had received therapy within 30 days of the ED visit. Patients visiting the ED for a non-cancer related reason and those with a new cancer diagnosis-related ED visit were given the highest proportion of the non-urgent triage category of 5 (30.8 and 34.6%, respectively, within the given group). Less than a third of the patients presenting at the ED for a non-cancer-related reason (30.1%) were hospitalized, and hospitalization was the lowest in this group, while patients with new cancer diagnosis-related ED visits had the highest percentage of hospitalization (60.8%) (Table 4).

Death within 30 days of ED admission was the highest among patients admitted due to cancer-related reasons and lowest among patients with a non-cancer related visit (46.7 and 5.1%, respectively; *p* = 0.000). The death rate within 30 days of ED admission of patients with oncological care-related ED visits was the second lowest (16.7%). The highest overall mortality in the study period was also found among patients with cancer-related ED visits (67.3%) and the lowest among patients with non-cancer-related ED visits (19.6%) (Table 4).

**Fig. 1:**
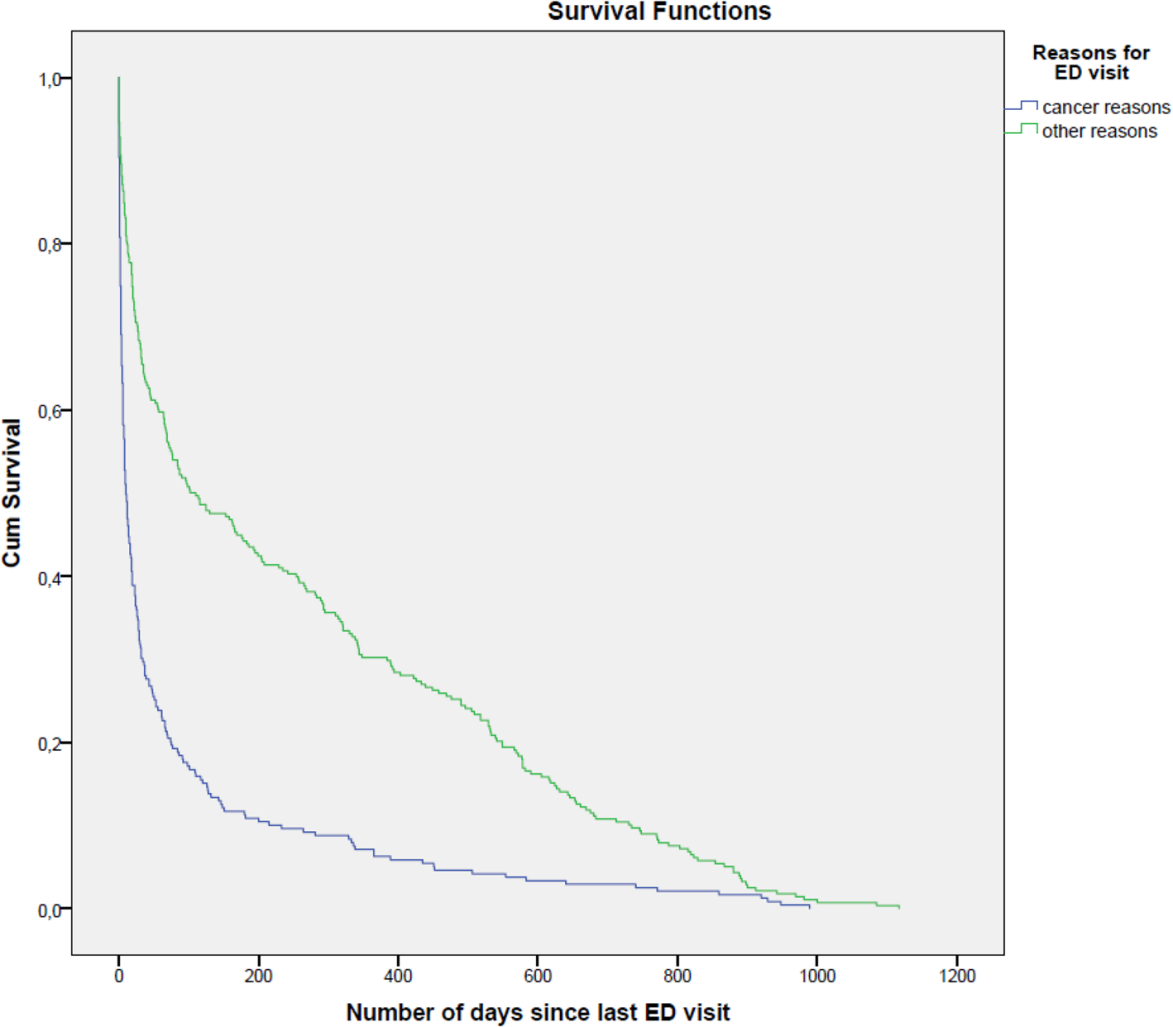
36-month overall survival comparing the two groups of patients with cancer based on reasons for the ED visit (1) cancer-related reason (2) non-cancer-related (other) reason

Similar observations were made when comparing patients’ 36-month overall survival, which showed that patients with cancer-related visits had a significantly worse survival outcome than patients with non-cancer related ED visits (Fig. 1).

Data regarding the distribution of the types of cancer, presenting symptoms, diagnosis codes and types of previous treatment are shown in Supplementary Tables 1A, 1B, 1C, 1D.

Colorectal cancer was significantly the most frequent type of cancer among patients with new cancer diagnosis-related ED visits (24.3%; *p* = 0.000), respiratory cancer among patients with a cancer-related ED visit (25.0%), and non-melanoma skin cancer along with breast cancer were most frequent among patients presenting with a non-cancer-related illness (16.0 and 15.2%, respectively) (Sup. 1A). Dyspnea, as a chief complaint was significantly more common among patients with a cancer-related ED visit (20.3%; *p* = 0.000), abdominal pain was most frequently found among patients with a new cancer diagnosis-related ED visit (20.3%), while patients with non-cancer-related illness tended to have the highest proportion of pain excluding headache, abdominal and chest pain (32.5%) (Sup. 1B). Gastrointestinal diseases were the most frequent diagnoses given following ED visit to patients with a new cancer diagnosis-related ED visit (29.1%), injuries and cardiovascular diseases as diagnoses were most frequent among patients with a non-cancer related visit (26.9 and 13.3%). Pain was most frequently registered as a diagnosis for patients with oncological care-related ED visits (14.1%). Gastrointestinal diseases and infections were equally the most common diagnoses given to patients whose reason for the ED visit was “undetermined” (11.9%) (Sup. 1C). Surgery, radiotherapy, and hormone treatment prior to the ED visit were comparatively the most common among patients visiting the ED for non-cancer-related reasons, while previous chemotherapy and immune/biological treatment were significantly most frequent among patients with an oncological care-related visit. Ongoing BSC/palliative care and hospice care were most common among patients with a cancer-related ED visit (Sup. 1D).

### 2. Predictive factors of cancer-related ED visits made by patients with cancer

We investigated whether predictive factors of cancer-related ED visits could be identified. Since cancer-related ED visits (23.2%) and non-cancer-related ED visits (58%), together comprised the majority (81.2%) of all ED visits made by patients with cancer, we analyzed the associations between the demographic and clinical parameters of patients visiting the ED for cancer-related and non-cancer related reasons.

**Fig. 2:**
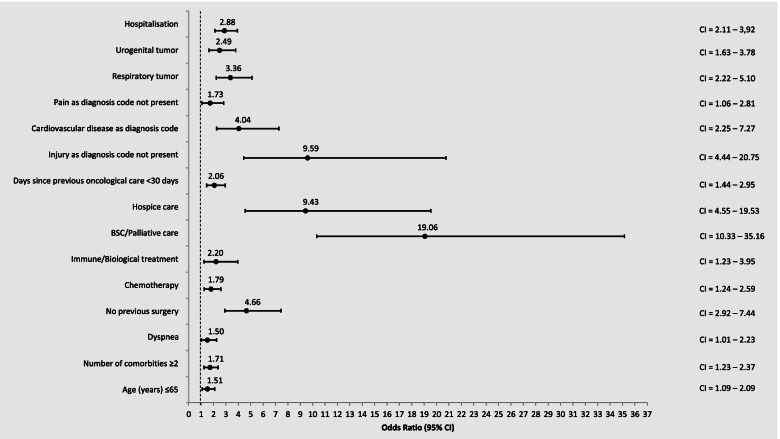
Independent predictive factors of the ED visit happening due to cancer-related reasons

Patients 65 years or below had an odds ratio of 1.51 and those with two or more comorbidities had an odds ratio of 1.71 for visiting the ED due to cancer-related reasons. Previous chemotherapy almost doubled, while previous oncological care within 30 days of ED presentation, immune/biological treatment, or the presence of a urogenital tumor more than doubled the odds of the ED visit being cancer-related (OR: 1.79, 2.06, 2.20 and 2.49, respectively). The presence of respiratory cancer or hospitalization of the patient approximately tripled, and the diagnosis of a cardiovascular disease quadrupled the odds of the ED visit being cancer-related (OR: 3.36, 2.88, and 4.04, respectively). No other chief complaint, except dyspnea (OR: 1.50), increased the odds of the ED visit to be due to cancer-related reasons. Patients currently receiving hospice care had significantly increased OR (OR: 9.43), however, the strongest predictor of a cancer- related ED visit was previous BSC/palliative care, with an OR of 19.06 (Fig. 2).

The lack of several factors, such as no previous surgical antitumor treatment or the lack of “injury” or “pain” as diagnosis codes also significantly influenced the odds of the ED visit being cancer- related (OR: 4.66, 9.59, and 1.73, respectively) (Fig. 2*).*

The other examined factors (e.g. patients’ sex, time of the ED visit, radiotherapy prior to the ED visit and presence of certain other types of cancers did not significantly affect the reason for the ED visit *(data not shown*).

## Discussion

Most studies investigating the characteristics of ED visits made by patients with cancer have focused on a single type of cancer [14–18], or have attempted to determine the reason for the ED visit by screening diagnosis codes given upon ED admission [2].

We analyzed the characteristics of patients with any type of cancer visiting the ED in one year and also identified predictive factors of cancer-related ED visits. By examining all cancer types, we have avoided the problem of some cancer-related visits being missed and ED visits that were not cancer-related from being mistakenly included.

The baseline ED visit rate at our ED Center was 27,010 visits made by adult patients, from which 21.4% were hospitalized in 2018. Compared to international data, where the hospitalization rates at the ED have been found to vary, between 12.4–58% depending on the studied country, such as the US, South-Korea, or Australia [10, 21, 22], our hospitalization rate was somewhere between these values. The observed differences between the countries are most probably due to the differences in the healthcare systems, and the distinct patient-routes of treatment. Unlike an Australian study reported, in Hungary there is no telephone triage, system or a ‘dedicated pathway’ for patients receiving oncological treatment. Therefore, according to the Australian study, patients may be admitted to EDs with more severe conditions, which could in turn result in significantly higher hospitalization rates [10]*.*

The proportion of patients with cancer visiting the ED in one year (8.8%) in our study is in line with previous investigations that have reported ED visit rates between 1 and 12% [3, 16, 23].

As in other studies, our results showed similar most common chief complaints, such as pain, dyspnea and nausea and/or vomiting [9, 24–26]. Our patients with cancer were older (above 65 years) and the majority had 2 or more comorbidities, which is similar to previous reports that have found that older patients were more likely to visit the ED [12, 27] and to have three comorbid conditions on average [9]. According to a large population-based study in the US, which investigated the reasons for ED visits by patients with cancer, the most common complaints patients with cancer mentioned upon ED presentation were related to pain, respiratory distress, and gastrointestinal issues, and that patients with lung, breast, prostate, and colorectal cancers were most likely to present at the ED [9]. In our study, we identified five main reasons of ED use by patients with cancer: cancer-related, oncological care-related, new cancer diagnosis-related, non-cancer related or undetermined ED visit. Each type of ED visit showed some distinct characteristics. Cancer-related ED visits were associated with the highest proportion of repeated ED visits and death within 30 days of ED admission. Cancer-related patient visits also had the most respiratory cancer cases, with leading chief complaint of dyspnea, the highest proportion of cases with prior BSC/palliative or hospice care, the most frequent ED visits per year and the lowest overall survival. Oncological care-related patients were younger, predominantly females, had received some form of oncological care/treatment 30 days prior to the ED visit, gastrointestinal diagnoses given following ED admission and previous chemo- or immune/biological therapy were most frequent among these patients. New cancer diagnosis-related cases were placed into the non-urgent triage level 5 category more often than patients from the other groups, yet they had the highest percentage of subsequent hospitalization; there were significantly more males in this group and colorectal cancer and abdominal pain as chief complaints were comparatively the most frequent among them. Non-cancer related ED visits were associated with patients who had the highest proportion of 2 or more comorbidities but had the lowest proportion of deaths within 30 days of the ED visit as well as highest OS. They have had previous surgery, radiotherapy or hormone treatment prior to the ED visit and had the lowest proportion of subsequent hospitalization. As expected, undetermined causes for ED visits showed a mixture of the characteristics of both cancer-related and non-cancer related ED visits.

Since we used a novel approach to categorizing the main reasons for ED visits, it is not possible to make direct comparisons with other studies. However, several previous investigations support our observations. In a study which analyzed unplanned ED visits by patients receiving oncological treatment found that receiving anti-cancer therapy 28 days prior to ED presentation was independently related to increased ED utilization [10], which is in accordance with our findings about oncological care-related ED visits. Furthermore, 78.3% of our oncological care-related ED cases had received some form of treatment, which was to be expected, considering that previous studies had also found high ED utilization ranging between 30 and 83% for patients having received chemotherapy [8, 24, 28, 29]. Also consistent with our results regarding cancer-related ED visits, Philip et al. found that patients with advanced cancer, who often require palliative care for chief complaints such as dyspnea and those with a limited life expectancy, frequently made ED visits for the management of their worsening symptoms due to the progression of their illness [30]. According to an investigation by Caterino et al., who analyzed the characteristics of patients with active cancer presenting to the ED, 62.5% of all patients had advanced or metastatic cancer and the third most common ED diagnosis among patients with active cancer was “abnormality in breathing [8]. In line with these results, our cases with cancer-related ED visits had a high proportion of respiratory cancer cases, with chief complaint of dyspnea and ongoing BSC/palliative or hospice care. In contrast, our non-cancer related visits often consisted of multimorbid patients presenting to the ED with relatively good prognosis and medical conditions independent of their cancer illness. Additionally, almost a third of these cases were given the non-urgent triage category of 5, indicating that these cases were not considered urgent by triage screening. As an earlier study found in a tertiary care ED center investigating non-urgent visits, over a quarter (25.8%) of patients cited “easier accessibility” and “limited resources and services” (17.8%) available in primary health care settings compared to EDs, as reasons for visiting the ED [31]. Furthermore, there has been an increasing trend in ED use among patients with chronic illnesses, which may partly be due to a shortage of primary care physicians [32]. These earlier observations may be the underlying reason for the majority of the ED visits occurring during off-clinic hours in our study. The comparatively high proportion of surgical treatment among our non-cancer related ED cases may be explained by the fact these patients’ tumors were probably still in an early stage, when complete surgical retreatment was still possible, therefore the patients’ prognoses were good. The low proportion of hospitalization and the lowest mortality rate within 30 days of ED presentation as well as during the follow-up period among the non-cancer related ED cases also support the previously mentioned hypothesis.

A recent study in the US found that 11% of new cancer diagnoses were ED-mediated and that these patients were discovered to have had colorectal cancer in 13% of the cases and to come from low socioeconomic backgrounds [33]. Although, it was not the main focus of our study, we found that our ED-mediated cancer diagnoses constituted 7.9% of all patients with cancer, they had a high proportion of subsequent hospitalization and a diagnosis of colorectal cancer. The higher proportion of newly diagnosed colorectal cancer among our cases may be due to the lack of a regular, nationwide colorectal screening program in Hungary.

To support our initial characterization of the main reasons for the ED visits we selected the two largest groups in our sample (cases of cancer-related ED visits vs non-cancer related ED visits) for further analysis, which together comprised the vast majority of all ED visits made by patients with cancer. We identified fifteen independent predictive factors of cancer-related ED visits. The younger age of the patient, having more than two comorbidities, dyspnea as chief complaint, respiratory cancer, prior chemotherapy, immune/biological treatment, BSC/palliative care, or hospice care, oncological treatment 30 days prior to the ED visit, hospitalization, and certain diagnoses (cardiovascular disease) or the lack of certain diagnoses (injury or pain), and no history of surgical antitumor treatment were all independent risk factors for a cancer-related ED visit.

In line with our results, a recent Canadian study showed that patients with lung cancers were significantly more likely to have had an ED visit compared to those diagnosed with other types of cancer, while chemotherapy, and immunotherapy were found to be less strongly associated with ED visits [34]. Also consistent with our findings, dyspnea as a chief complaint for the ED visit was found to be very common, between the second and fourth main reasons for an ED visit [35], which phenomenon may possibly indicate the progression of the cancer and worse prognosis [4, 36]. A previous study reported that factors associated with approaching death were lung cancer and dyspnea among other factors [37], which may explain the high death rate and decreased overall survival we observed among our patients visiting the ED for cancer-related reasons. Hospitalization following ED presentation also significantly increased the odds of our patients’ visit to be cancer related. Interestingly, a study conducted in the Netherlands also showed that patients with metastasized colon, bronchus, or lung cancer received a significant amount of in-hospital medical care (up to 65%), including hospital and ED admissions, and that specialized palliative care was initiated relatively late [38]. Other studies reported similar results, with hospitalization rates of 76% in advanced care patients [37] and 58% across all oncology patients [25]. With specialized palliative care not being readily available to all patients and distributed unevenly across Hungary, the high ORs of our patients receiving hospice care and particularly BSC/palliative care may indicate the unmet supportive care needs of these patient groups. Furthermore, since patients with advanced cancer - who often receive palliative or hospice care- have progressing symptoms, it was to be expected that their ED visits would often be cancer-related. Our findings emphasize the distinct characteristics of the patients receiving palliative/hospice care within the cancer-related cases. In patients with colon cancer, ED encounters within 30 days of surgery discharge were common [14] and another analysis also indicated that recently hospitalized patients often visited the ED for care [39]. In our population of patients with different types of tumors, we found similar results: oncological treatment 30 days prior to the ER visit increased the odds of an ED visit being cancer related. According to some earlier studies older age may be a predictor of ED use by patients with cancer [11, 13]. In contrast, we found that younger age increased the odds of the ED visit being cancer related. These apparently different results may be explained by the different methodologies of data analysis, since we focused on a population with all types of cancers but only those with directly cancer-related reasons for the ED visit, while most other studies focused on specific types of patients with cancer with less focus on the main reasons for the ED visit.

By analyzing the ED visits of patients with all types of cancer, we provided a snapshot of the characteristics of patients with cancer visiting the ED, which thus, may have practical implications for healthcare staff at the ED. Visits of patients with cancer constitute a relevant proportion of all ED visits and while a part of these visits are related to the progression or previous treatment of the patient, a number of patients have medical conditions completely unrelated to their cancer illness and are nonurgent. Determining which group the patient belongs to and allocating the required healthcare resources, may be aided by being aware of the predictors of directly cancer-related conditions. The burdens of ED healthcare staff would be alleviated if medical conditions related to the progression or complications of the cancer disease could be attended to by special cancer support initiatives, as implemented in some models-of-care in the US, UK, and Canada [34, 40].

## Limitations

Our study has limitations. The study was conducted at a single site tertiary level ED, therefore further investigations need to be carried out in multiple sites to confirm our results. By choosing to provide an overview of patients with all types of cancer, we could not consider the characteristics of special cancer types or the cancer being active or not, which may influence ED visit frequency and hospital admission. Furthermore, the classification of certain data (symptoms, types of cancer, etc.) into larger categories could have led to classification bias. Although patients treated at our hospital are required to present to our ED Center if they develop urgent symptoms, some patients may have been admitted to another ED center if they were in another location (other city, county or even country) when they developed urgent symptom, thus limiting the inclusion of all patients’ all ED visits in our study. These limitations of our study may therefore have influenced our results.

## Conclusions

To our knowledge this is the first study to characterize the main causes of ED visits in a general cancer population at a tertiary-level care clinic from the Central-Eastern European region. It is also the first to identify independent predictors of ED use by patients with cancer - using a novel approach - based on the main cause (chief complaint) of their visit. We demonstrated that approximately one-fourth of the general cancer population visited the ED due to the progression or complications of their cancer disease and that these patients had a shorter overall survival than other patients with cancer. We also found that younger age, chief complaint of dyspnea, respiratory cancer, prior chemotherapy, immune/biological treatment, BSC/palliative care, or hospice care, oncological treatment 30 days prior to the ED visit, hospitalization and certain diagnoses given following ED admission were independent risk factors for a cancer-related ED visit. By gathering information about the predisposing factors of ED visits made by patients with cancer, there is a possibility for identifying risk groups, providing focused patient education, and other forms of non-emergent care.

Our results may provide important information for the development of algorithms intended to identify the needs of care of patients with cancer at the ED.

## Supplementary Information

**Additional file 1: Supplementary Table 1A**. Types of cancer based on the reason for the ED visit.

**Additional file 2: Supplementary Table 1B**. Presenting symptoms based on the reason for the ED visit.

**Additional file 3: Supplementary Table 1C**. Diagnosis codes following ED presentation based on the reason for the ED visit.

**Additional file 4: Supplementary Table 1D**. Types of previous oncological care of patients based on the reason for the ED visit.

## Data Availability

The datasets generated and/or analyzed during the current study are not publicly due to data privacy of human patients but are available from the corresponding author on reasonable request.

## Abbreviations

ED: Emergency Department
CTAS: Canadian Triage and Acuity Scale
MSTR: Magyar Sürgősségi Triage Rendszer = Hungarian Emergency Triage System
ICD-10: International Classification of Diseases, 10th Revision
COPD: Chronic Obstructive Pulmonary Disease
BSC: Best Supportive Care
OR: Odds Ratio
OS: Overall Survival
