# Supplementary material for: Main reasons and predictive factors of cancer-related emergency department visits in a Hungarian tertiary care center
Source: BMC Emerg Med. 2022 Jun 23;22:114. doi: 10.1186/s12873-022-00670-0 (PMC9219147; doi:10.1186/s12873-022-00670-0)
Supplement: Supplementary file 1 — Additional file 1: Supplementary Table 1A. Types of cancer based on the reason for the ED visit. [file 12873_2022_670_MOESM1_ESM.docx]

|  | Cancer-related ED visit  n=552 | Oncological care-related ED visit  n=85 | New cancer diagnosis -related ED visit  n=189 | Non-cancer related ED visit n=1381 | Undetermined ED visit  n=176 | Total number of ED visits by cancer patients  N=2383 | p |
| --- | --- | --- | --- | --- | --- | --- | --- |
| Colorectal cancer | 91  (16.5%) | 19  (22.4%) | 46  (24.3%) | 199  (14.4%) | 37  (21.0%) | 392  (16.4%) | 0.000 |
| Breast cancer | 34  (6.2%) | 7  (8.2%) | 6  (3.2%) | 210  (15.2%) | 18  (10.2%) | 275  (11.5%) |  |
| Gastro-esophageal cancer | 28  (5.1%) | 3  (3.5%) | 16  (8.5%) | 32  (2.3%) | 8  (4.5%) | 87  (3.7%) |  |
| Urogenital cancer (excepting prostate) | 81  (14.7%) | 12  (14.1%) | 14  (7.4%) | 189  (13.7%) | 29  (16.5%) | 325  (13.6%) |  |
| Prostate  cancer | 31  (5.6%) | 6  (7.1%) | 7  (3.7%) | 137  (9.9%) | 20  (11.4%) | 201  (8.4%) |  |
| Head and Neck cancer | 22  (4.0%) | 3  (3.5%) | 4  (2.1%) | 53  (3.8%) | 3  (1.7%) | 85  (3.6%) |  |
| Pancreas, small intestine, liver, gallbladder, biliary tract cancer | 68  (12.3%) | 2  (2.4%) | 25  (13.2%) | 33  (2.4%) | 7  (4.0%) | 135  (5.7%) |  |
| Respiratory cancer | 138  (25.0%) | 23  (27.1%) | 29  (15.3%) | 118  (8.5%) | 33  (18.8%) | 341  (14.3%) |  |
| Hematological cancer | 11  (2.0%) | 4  (4.7%) | 11  (5.8%) | 82  (5.9%) | 11  (6.3%) | 119  (5.0%) |  |
| Melanoma | 3  (0.5%) | 1  (1.2%) | 2  (1.1%) | 44  (3.2%) | 0  (0.0%) | 50  (2.1%) |  |
| Other non-melanoma skin cancer | 1  (0.2%) | 1  (1.2%) | 4  (2.1%) | 221  (16.0%) | 2  (1.1%) | 229  (9.6%) |  |
| Other cancers | 44  (8.0%) | 4  (4.7%) | 25  (13.2%) | 63  (4.6%) | 8  (4.5%) | 144  (6.0%) |  |

**Supplementary Table 1A: Types of cancer based on the reason for the ED visit**
