# Supplementary material for: Main reasons and predictive factors of cancer-related emergency department visits in a Hungarian tertiary care center
Source: BMC Emerg Med. 2022 Jun 23;22:114. doi: 10.1186/s12873-022-00670-0 (PMC9219147; doi:10.1186/s12873-022-00670-0)
Supplement: Supplementary file 2 — Additional file 2: Supplementary Table 1B. Presenting symptoms based on the reason for the ED visit. [file 12873_2022_670_MOESM2_ESM.docx]

|  | Cancer-related ED visit  n=552 | Oncological care -related ED visit  n=85 | New cancer diagnosis -related ED visit  n=189 | Non-cancer related ED visit  n=1381 | Undetermined ED visit  n=176 | Total number of ED visits by cancer patients  N=2383 | p |
| --- | --- | --- | --- | --- | --- | --- | --- |
| Complaints related to the skin or tissues below the skin | 11  (2.0%) | 3  (3.5%) | 5  (2.6%) | 26  (1.9%) | 5  (2.8%) | 50  (2.1%) | 0.000 |
| Dyspnea | 112  (20.3%) | 10  (11.8%) | 31  (16.4%) | 151  (10.9%) | 17  (9.7%) | 321  (13.5%) |  |
| Other gastrointestinal complaints | 29  (5.3%) | 12  (14.1%) | 25  (13.2%) | 45  (3.3%) | 9  (5.1%) | 120  (5.0%) |  |
| Other pain (excluding headache, chest- and abdominal pain) | 31  (5.6%) | 3  (3.5%) | 18  (9.5%) | 449  (32.5%) | 20  (11.4%) | 521  (21.9%) |  |
| Other complaints related to the nervous system | 24  (4.3%) | 0  (0.0%) | 13  (6.9%) | 49  (3.5%) | 9  (5.1%) | 95  (4.0%) |  |
| Other nondefined complaints | 14  (2.5%) | 2  (2.4%) | 3  (1.6%) | 28  (2.0%) | 19  (10.8%) | 66  (2.8%) |  |
| Headache | 6  (1.1%) | 0  (0.0%) | 1  (0.5%) | 36  (2.6%) | 4  (2.3%) | 47  (2.0%) |  |
| Nausea and vomiting | 54  (9.8%) | 14  (16.5%) | 17  (9.0%) | 49  (3.5%) | 16  (9.1%) | 150  (6.3%) |  |
| Abdominal pain | 86  (15.6%) | 9  (10.6%) | 38  (20.1%) | 141  (10.2%) | 35  (19.9%) | 309  (13.0%) |  |
| Disturbances of cognition and vigility | 17  (3.1%) | 1  (1.2%) | 8  (4.2%) | 31  (2.2%) | 2  (1.1%) | 59  (2.5%) |  |
| Fever | 9  (1.6%) | 7  (8.2%) | 1  (0.5%) | 21  (1.5%) | 5  (2.8%) | 43  (1.8%) |  |
| Complaints related to the Respiratory system | 16  (2.9%) | 2  (2.4%) | 5  (2.6%) | 36  (2.6%) | 3  (1.7%) | 62  (2.6%) |  |
| Chest pain | 21  (3.8%) | 4  (4.7%) | 7  (3.7%) | 88  (6.4%) | 4  (2.3%) | 124  (5.2%) |  |
| Psychiatric complaints | 1  (0.2%) | 0  (0.0%) | 0  (0.0%) | 13  (0.9%) | 0  (0.0%) | 14  (0.6%) |  |
| Malaise, fatigue | 24  (4.3%) | 6  (7.1%) | 5  (2.6%) | 18  (1.3%) | 7  (4.0%) | 60  (2.5%) |  |
| Fainting, collapsing | 17  (3.1%) | 2  (2.4%) | 2  (1.1%) | 45  (3.3%) | 5  (2.8%) | 71  (3.0%) |  |
| Vertigo | 9  (1.6%) | 1  (1.2%) | 3  (1.6%) | 45  (3.3%) | 0  (0.0%) | 58  (2.4%) |  |
| Uneven heartbeat | 4  (0.7%) | 1  (1.2%) | 0  (0.0%) | 34  (2.5%) | 3  (1.7%) | 42  (1.8%) |  |
| Feeding problems | 17  (3.1%) | 2  (2.4%) | 1  (0.5%) | 8  (0.6%) | 0  (0.0%) | 28  (1.2%) |  |
| Bleeding | 14  (2.5%) | 3  (3.5%) | 2  (1.1%) | 0  (1.4%) | 0  (0.0%) | 39  (1.6%) |  |
| Difficulty with urinating | 36  (6.5%) | 3  (3.5%) | 4  (2.1%) | 48  (3.5%) | 13  (7.4%) | 104  (4.4%) |  |

**Supplementary Table 1B: Presenting symptoms based on the reason for the ED visit.**
