# Supplementary material for: Main reasons and predictive factors of cancer-related emergency department visits in a Hungarian tertiary care center
Source: BMC Emerg Med. 2022 Jun 23;22:114. doi: 10.1186/s12873-022-00670-0 (PMC9219147; doi:10.1186/s12873-022-00670-0)
Supplement: Supplementary file 3 — Additional file 3: Supplementary Table 1C. Diagnosis codes following ED presentation based on the reason for the ED visit. [file 12873_2022_670_MOESM3_ESM.docx]

|  | Cancer-related ED visit  n=552 | Oncological care -related ED visit  n=85 | New cancer diagnosis -related ED visit  n=189 | Non-cancer related ED visit  n=1381 | Undetermined ED visit  n=176 | Total number of ED visits by cancer patients  N=2383 | p |
| --- | --- | --- | --- | --- | --- | --- | --- |
| Allergy | 0  (0.0%) | 2  (2.4%) | 1  (0.5%) | 5  (0.4%) | 0  (0.0%) | 8  (0.3%) | 0.000 |
| Other general complaints | 12  (2.2%) | 2  (2.4%) | 3  (1.6%) | 15  (1.1%) | 5  (2.8%) | 37  (1.6%) |  |
| Skin and soft tissue diseases | 3  (0.5%) | 1  (1.2%) | 0  (0.0%) | 8  (0.6%) | 0  (0.0%) | 12  (0.5%) |  |
| Cardiovascular diseases | 21  (3.8%) | 7  (8.2%) | 13  (6.9%) | 183  (13.3%) | 18  (10.2%) | 242  (10.2%) |  |
| Bone, muscle and connective tissue diseases | 6  (1.1%) | 2  (2.4%) | 4  (2.1%) | 44  (3.2%) | 7  (4.0%) | 63  (2.6%) |  |
| Cancer diseases | 36  (6.5%) | 8  (9.4%) | 27  (14.3%) | 9  (0.7%) | 9  (5.1%) | 89  (3.7%) |  |
| Endocrinological diseases | 2  (0.4%) | 0  (0.0%) | 0  (0.0%) | 11  (0.8%) | 1  (0.6%) | 14  (0.6%) |  |
| Pain | 45  (8.2%) | 12  (14.1%) | 25  (13.2%) | 175  (12.7%) | 18  (10.2%) | 275  (11.5%) |  |
| Infections | 51  (9.2%) | 6  (7.1%) | 18  (9.5%) | 135  (9.8%) | 21  (11.9%) | 231  (9.7%) |  |
| Fluid loss and electrolyte disturbances | 51  (9.2%) | 4  (4.7%) | 10  (5.3%) | 45  (3.3%) | 11  (6.3%) | 121  (5.1%) |  |
| ENT-, and oral cavity diseases | 0  (0.0%) | 0  (0.0%) | 0  (0.0%) | 4  (0.3%) | 1  (0.6%) | 5  (0.2%) |  |
| Gastrointestinal diseases | 116  (21.0%) | 22  (25.9%) | 55  (29.1%) | 104  (7.5%) | 21  (11.9%) | 318  (13.3%) |  |
| Fever | 6  (1.1%) | 1  (1.2%) | 0  (0.0%) | 9  (0.7%) | 3  (1.7%) | 19  (0.8%) |  |
| Airways and breathing disorders | 80  (14.5%) | 4  (4.7%) | 9  (4.8%) | 65  (4.7%) | 8  (4.5%) | 166  (7.0%) |  |
| Mental and behavioral disturbances | 5  (0.9%) | 0  (0.0%) | 1  (0.5%) | 13  (0.9%) | 0  (0.0%) | 19  (0.8%) |  |
| Poisoning | 4  (0.7%) | 0  (0.0%) | 0  (0.0%) | 11  (0.8%) | 0  (0.0%) | 15  (0.6%) |  |
| Neurological diseases | 21  (3.8%) | 2  (2.4%) | 7  (3.7%) | 59  (4.3%) | 8  (4.5%) | 97  (4.1%) |  |
| Injuries | 15  (2.7%) | 1  (1.2%) | 8  (4.2%) | 371  (26.9%) | 5  (2.8%) | 400  (16.8%) |  |
| No illness | 0  (0.0%) | 0  (0.0%) | 0  (0.0%) | 7  (0.5%) | 5  (2.8%) | 12  (0.5%) |  |
| Triage referral | 16  (2.9%) | 4  (4.7%) | 0  (0.0%) | 20  (1.4%) | 12  (6.8%) | 52  (2.2%) |  |
| Diseases of the urogenital system | 36  (6.5%) | 3  (3.5%) | 4  (2.1%) | 56  (4.1%) | 11  (6.3%) | 110  (4.6%) |  |
| Vascular diseases | 11  (2.0%) | 0  (0.0%) | 0  (0.0%) | 17  (1.2%) | 4  (2.3%) | 32  (1.3%) |  |
| Blood cell disorders | 11  (2.0%) | 4  (4.7%) | 4  (2.1%) | 8  (0.6%) | 7  (4.0%) | 34  (1.4%) |  |
| Bleeding | 4  (0.7%) | 0  (0.0%) | 0  (0.0%) | 7  (0.5%) | 1  (0.6%) | 12  (0.5%) |  |

**Supplementary Table 1C: Diagnosis codes following ED presentation based on the reason for the ED visit**
