# Supplementary material for: Main reasons and predictive factors of cancer-related emergency department visits in a Hungarian tertiary care center
Source: BMC Emerg Med. 2022 Jun 23;22:114. doi: 10.1186/s12873-022-00670-0 (PMC9219147; doi:10.1186/s12873-022-00670-0)
Supplement: Supplementary file 4 — Additional file 4: Supplementary Table 1D. Types of previous oncological care of patients based on the reason for the ED visit. [file 12873_2022_670_MOESM4_ESM.docx]

|  | Cancer-related ED visit  n=552 | | Oncological care -related ED visit  n=85 | New cancer diagnosis -related ED visit  n=189 | Non-cancer related ED visit n=1381 | Undetermined ED visit  n=176 | Total number of ED visits by cancer patients  N=2383 | p |
| --- | --- | --- | --- | --- | --- | --- | --- | --- |
| Surgical treatment | | 35  (6.3%) | 15  (17.6%) | 8  (4.2%) | 523  (37.9%) | 28  (15.9%) | 609  (25.6%) | 0.000 |
| Radiotherapy | | 82  (14.9%) | 8  (9.4%) | 4  (2.1%) | 222  (16.1%) | 24  (13.6%) | 340  (14.3%) | 0.000 |
| Chemotherapy | | 134  (24.3%) | 48  (56.5%) | 10  (5.3%) | 227  (16.4%) | 57  (32.4%) | 476  (20.0%) | 0.000 |
| Immuno/ biological therapy | | 35  (6.3%) | 8  (9.4%) | 2  (1.1%) | 56  (4.1%) | 12  (6.8%) | 113  (4.7%) | 0.003 |
| Hormone therapy | | 27  (4.9%) | 6  (7.1%) | 3  (1.6%) | 188  (13.6%) | 8  (4.5%) | 232  (9.7%) | 0.000 |
| BSC/palliative care | | 107  (19.4%) | 1  (1.2%) | 3  (1.6%) | 22  (1.6%) | 2  (1.1%) | 135  (5.7%) | 0.000 |
| Hospice care | | 64  (11.6%) | 0  (0.0%) | 4  (2.1%) | 15  (1.1%) | 7  (4.0%) | 90  (3.8%) | 0.000 |

**Supplementary Table 1D: Types of previous oncological care of patients based on the reason for the ED visit**
